# More Than Just a Removal Service: Scavenger Receptors in Leukocyte Trafficking

**DOI:** 10.3389/fimmu.2018.02904

**Published:** 2018-12-12

**Authors:** Daniel A. Patten, Shishir Shetty

**Affiliations:** National Institute for Health Research Birmingham Liver Biomedical Research Unit and Centre for Liver and Gastrointestinal Research, Institute of Immunology and Immunotherapy, University of Birmingham, Birmingham, United Kingdom

**Keywords:** leukocyte adhesion cascade, SR-AI, LOX-1, mannose receptor, SCARF1, SR-PSOX, stabilin-1, stabilin-2

## Abstract

Scavenger receptors are a highly diverse superfamily of proteins which are grouped by their inherent ability to bind and internalize a wide array of structurally diverse ligands which can be either endogenous or exogenous in nature. Consequently, scavenger receptors are known to play important roles in host homeostasis, with common endogenous ligands including apoptotic cells, and modified low density lipoproteins (LDLs); additionally, scavenger receptors are key regulators of inflammatory diseases, such as atherosclerosis. Also, as a consequence of their affinity for a wide range of microbial products, their role in innate immunity is also being increasingly studied. However, in this review, a secondary function of a number of endothelial-expressed scavenger receptors is discussed. There is increasing evidence that some endothelial-expressed scavenger receptors are able to directly bind leukocyte-expressed ligands and subsequently act as adhesion molecules in the trafficking of leukocytes in lymphatic and vascular tissues. Here, we cover the current literature on this alternative role for endothelial-expressed scavenger receptors and also speculate on their therapeutic potential.

## Introduction

The first scavenger receptor was described in the late 1970s by Brown and Goldstein and was defined by its ability to bind and subsequently internalize low density lipoproteins (LDLs) (1, 2). However, the term “scavenger receptor” was not coined until a couple of years later in the early 1980s by Fogelman et al. who were studying the functionality of Brown and Goldstein's LDL receptor in monocytes and macrophages (3). Scavenger receptors are now a large superfamily of proteins which are highly diverse in structure and are sub-divided into a number of classes (class A-J), with each class sharing structural features; however, there is little or no sequence homology between the classes and the superfamily grouping is purely a consequence of shared functional properties (4). Functionally, scavenger receptors have an important role in both homeostatic and disease states, as they detect and remove, or scavenge, unsolicited self-antigens, which predominantly manifest as damage-associated molecular patterns (DAMPs), such as phosphatidylserine on apoptotic cells (5–7) and products of oxidative stress (e.g., oxidized (ox)LDLs) (8, 9), from general circulation. The removal of apoptotic host cells by scavenger receptors is particularly pertinent in the context of autoimmune diseases, such as systemic lupus erythematosus (SLE), which has been shown to spontaneously develop in some lines of scavenger receptor-deficient mice (7, 10), thus highlighting their role in homeostasis. Also, other clinical manifestations, for example severe renal glomerular fibrosis and premature mortality, have been shown to spontaneously develop in some multiple scavenger receptor-deficient mice as a result of impaired clearance of harmful factors, such as growth differentiation factor (GDF)-15, from the systemic blood supply (11). These severe phenotypes are somewhat surprising given that several scavenger receptors are able to bind a number of common ligands; therefore, one would assume there would be a certain amount of redundancy in their function and, in the absence of one scavenger receptor, the others would be up-regulated in a compensatory manner to maintain homeostasis. Nevertheless, this is clearly not the case for several members of the scavenger superfamily.

In a number of murine models of inflammatory diseases, the lack of certain scavenger receptors has been shown to be highly detrimental, thus implicating these receptors in the limitation of disease pathology. For example, in a murine model of Alzheimer's disease, reduction or deletion of scavenger receptor class B type I (SR-BI) resulted in increased severity of disease due to impaired clearance of amyloid-β by infiltrating macrophages (12). More recently, we have shown that a lack of the class H scavenger receptor, stabilin-1, in murine models of liver injury promotes fibrogenesis, due to impaired clearance of malondialdehyde (MDA) modified oxLDLs (MDA-LDLs) (13). Conversely, some scavenger receptors have been shown to actively contribute to disease pathology, with several implicated in the establishment, and progression of atherosclerosis due their role in the uptake and storage of LDLs in macrophages (14–17). Furthermore, scavenger receptors also play an important role in the host innate immune system (18–21), as the majority of scavenger receptors are differentially expressed in a number of professional innate immune cells, such as monocytes, macrophages and dendritic cells (22, 23), and are able to recognize a huge array of microbial antigens (24, 25). However, the paradigm is now being established that scavenger receptors require the presence of other pattern recognition receptors (PRRs), such as Toll-like receptors (TLRs), in order to elicit an immunological response (26–30).

In addition to their intrinsic scavenging capacity, a number of endothelial-expressed scavenger receptors also exhibit a secondary function in host immunity as they are able to directly interact with leukocytes and mediate their passage across a range of endothelia. This secondary function has led to the study of some scavenger receptors in lymphocyte migration in lymph nodes and in the extravasation of leukocytes during inflammation. In this review, we initially discuss the processes of leukocyte trafficking, subsequently explore the current knowledge of scavenger receptor involvement in these processes and speculate on future research and potential for this relatively understudied function of scavenger receptors.

### Lymphocyte Trafficking in Lymph Nodes

The antigen-driven adaptive immune system requires regulated trafficking of T cells in order to orchestrate lymphocyte development, immune surveillance, rapid immunological responses, and memory (31). Consequently, lymphocytes are continually recirculating between the vascular and lymphatic systems and organ tissues. T cells which have not previously encountered antigens, termed naïve T cells, are programmed to undergo migratory cycles into and out of secondary lymphoid organs (SLOs), such as peripheral lymph nodes, tonsils, and Peyers patches, in search of cognate antigens (31). T cells enter lymph nodes (LNs) through afferent lymphatic vessels or high endothelial venules (HEVs) (32) and subsequently interact with antigen presenting cells, primarily dendritic cells (DCs), which present antigens encountered in inflamed tissues on their surface via major histocompatibility complex (MHC) proteins (33). Once T cells encounter cognate MHC/antigen, in concert with the relevant co-stimulatory or co-inhibitory molecules, they become activated, and undergo differentiation into antigen-specific effector or memory cells (33). The trafficking of T cells to and from lymph nodes is known to involve intimate interactions with lymphatic endothelial cells (LECs); however, the endothelial-expressed molecules involved in these processes are not well characterized (31). Nevertheless, the involvement of scavenger receptors has been suggested and is discussed throughout this review.

### The Leukocyte Adhesion Cascade

**Figure 1:**
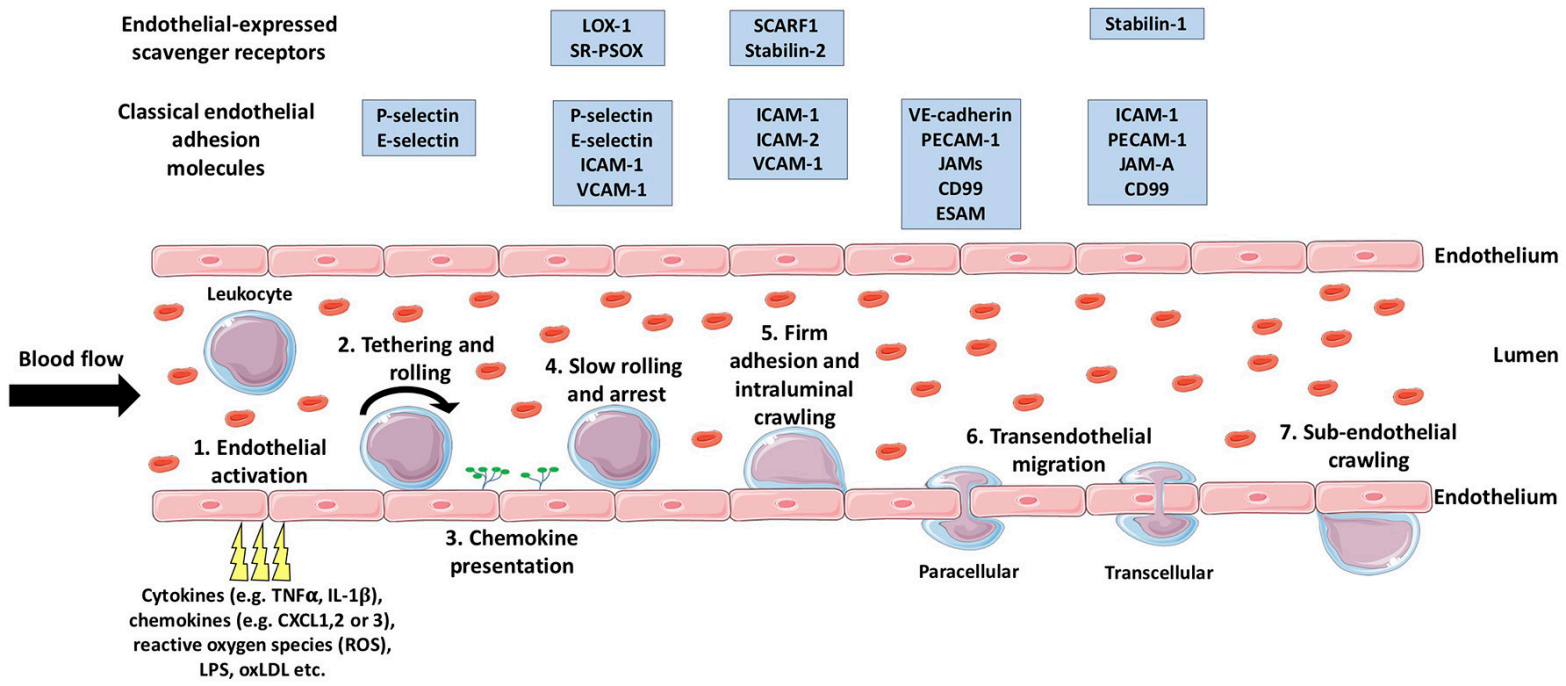
The multistep leukocyte adhesion cascade. Leukocytes are recruited from the bloodstream to inflamed tissues via sequential multi-step process known as the leukocyte adhesion cascade. Firstly, endothelial activation is triggered by a range of endogenous or exogenous stimuli from the inflamed tissue (1), which triggers the selectin-dependent tethering and rolling of leukocytes along the luminal surface of the vessel (2). Subsequently, chemokines are presented on the luminal surface of the endothelium (3) which activate leukocyte-expressed integrins allowing stronger bond formation with their endothelial-expressed ligands. The formation of these stronger leukocyte-endothelium bonds results in leukocyte arrest (4), following which, intraluminal crawling occurs (5). Next, the leukocyte will undergo transendothelial migration (6) either via the paracellular or the transcellular pathway. Once the leukocyte has crossed the endothelial layer, it may undertake in sub-endothelial crawling (7), prior to entering the target tissue proper. TNFα, tumor necrosis factor-α; IL-1β, interleukin-1β; LPS, lipopolysaccharide; oxLDL, oxidized low density lipoprotein; LOX-1, Lectin-like oxidized low-density lipoprotein receptor-1; SR-PSOX, scavenger receptor that binds phosphatidylserine and oxidized lipids; ICAM-1, intercellular adhesion molecule-1; VCAM-1, vascular cell adhesion molecule-1 ; SCARF1, scavenger receptor class F; member 1; VE-cadherin, vascular endothelial cadherin; PECAM-1, platelet endothelial cell adhesion molecule-1; JAMs, junctional adhesion molecules; ESAM, endothelial cell-specific adhesion molecule. (Stock images provided by Servier medical for use under the Creative Commons Attribution 3.0 Unported License).

During injury or infection, leukocytes in the blood are required to migrate from general circulation, across the vascular endothelium, and into the inflamed tissue, with the primary aim of eliminating the inflammatory trigger and/or contributing to tissue repair (34). In general, the migration of leukocytes from the blood into inflamed tissues occurs in post-capillary venules, with the exception of the liver, spleen and lungs (34). Leukocyte migration is achieved via a multi-step process known as the leukocyte adhesion cascade (35) (Figure 1), in which the leukocytes initially tether and roll on the luminal surface of the blood vessel and undergo arrest, followed by firm adhesion and, finally, migrate through the endothelial barrier into the tissue (36). This sequence of events is mediated by a large number of chemoattractant cytokines (chemokines) (37) and adhesion molecules (Figure 1) which determine the subset of leukocyte to be recruited to the site of inflammation and subsequently regulate their numbers (34). Additionally, crossing the vascular wall is not only a highly selective and regulatory step in leukocyte migration, but also acts to prime the tissue-infiltrating leukocytes (38) in order to deliver an efficient and effective immunological response.

#### Endothelial Activation, Initial Capture, and Rolling

Endothelial activation is the initial step which results in the expression of adhesion molecules and chemokines on the luminal membrane of endothelial cells involved in the initial capture of leukocytes from shear flow. Endothelial activation can be triggered by a wide range of stimuli and is classified as “type I” or “type II,” depending on the mediating signal molecule. Type I activation of endothelial cells is a protein-synthesis-independent process and is predominantly mediated via ligands of heterotrimeric G-protein-coupled receptors (GPCRs), such as histamine and thrombin (39). Type I activation results in the trafficking of pre-formed P-selectin to the cell membrane within minutes, thus allowing the rapid recruitment of neutrophils to vascular endothelia (40–43). Type I activation is a highly transient event and, in order to limit the extent of neutrophil extravasation, the GPCRs involved are presumed to undergo desensitization (44, 45) to their stimuli after 10–20 min to prevent further endothelial stimulation (39). Type II activation of endothelial cells is a much slower process known to be triggered by a much wider range of stimuli, including inflammatory cytokines [e.g., tumor necrosis factor (TNF)α, interferon (IFN)γ, and interleukin (IL)-1β (46)], microbial antigens [e.g., lipopolysaccharide (LPS) (47, 48)], and oxLDLs (49, 50). Type II activation results in morphological changes, via the reorganization of actin filaments (51) and *de novo* expression of leukocyte adhesion molecules, such as E-selectin, intracellular adhesion molecule (ICAM)-1 and vascular cell-adhesion molecule (VCAM)-1 (52–55), and chemokines (56, 57) on the luminal surface of the endothelial cells. Unlike, type I activation which is stringently regulated via receptor desensitization, type II activation is much more long-lived and can chronically persist until the inflammatory stimulus is removed and the regulatory anti-inflammatory feedback mechanisms are able to effectively counteract the proinflammatory exacerbation, commonly via regulation of the nuclear factor (NF)-κB pathway (58, 59).

Following endothelial activation, the initial capture of leukocytes from shear flow is mediated by selectins, a family of three Type I transmembrane Ca^2+^-dependent lectins which bind to glycoprotein ligands (60). The selectins are named according to the cell type in which they were originally described in (platelet (P)-selectin, leukocyte (L)-selectin and endothelial (E)-selectin) and consist of an extracellular N-terminal C-type lectin domain, an epidermal growth factor (EGF)-like domain, a series of short consensus repeats (SCRs), a transmembrane domain and a short C-terminal intracellular domain (61). As mentioned above, stores of pre-formed P-selectin are held within human endothelial cells (62) and are rapidly trafficked to the surface in the event of type I activation, but P-selectin is also differentially regulated in a range of chronic inflammatory diseases (63–66) and plays a major role in leukocyte recruitment during prolonged type II activation (67–70). L-selectin is expressed in the majority of circulating leukocytes and is one of the first leukocyte-expressed cell adhesion molecules to interact with the endothelial layer in the initial “tethering” event (71), whereas E-selectin is constitutively expressed in bone marrow endothelial cells (72), but is inducible in other endothelia (54). E-selectin is predominantly involved in the rolling and slow rolling steps of the adhesion cascade (73, 74). Rolling is the transient and reversible selectin-ligand interaction which involves the “catch-bond” phenomenon, where bonds are strengthened with increasing shear stress (75). Also, the rolling motion of leukocytes is able to generate new selectin-ligand bonds before old ones are broken via the “tether and sling” phenomenon utilized by neutrophils (76, 77) and differentiated T cell subsets (78). The rolling and slow rolling steps aim to initiate leukocyte-endothelial contact and, consequently, further activate the leukocyte, thus promoting the successive steps in the adhesion cascade.

#### Leukocyte Arrest and Crawling

The arrest of leukocytes rolling along the surface of the endothelium is triggered by chemokines which are expressed upon endothelial activation and are immobilized on the luminal surface via highly negatively-charged polysaccharides, such as glycosaminoglycans (GAGs) (79, 80). As a consequence of chemokine-induced “inside-out” signaling, heterodimeric adhesion receptors expressed on the surface of leukocytes, known as integrins, undergo conformational changes and become “activated” (81, 82). Once activated, integrins are able to form high affinity bonds with their endothelial-expressed ligands and their clustering in focal adhesion contacts allows for stronger leukocyte-endothelial bonds (83), thus resulting in leukocyte arrest [reviewed in detail by Ley et al. (35)].

Once firmly adhered to the endothelial layer, innate immune cells, such as monocytes, have been shown to patrol the vessel wall surface (84), scavenging microparticles, and supporting the recruitment of other cells, such as neutrophils (85). This intraluminal “crawling” behavior has also been observing in neutrophils and is thought to mediate their transmigration across the endothelial layer, as they search for sites of exit from the blood vessel (86–88). Additionally, a novel phenomenon in hepatic sinusoidal endothelial cells (HSEC) was recently described in which peripheral blood lymphocytes were shown to migrate horizontally from one endothelial cell to another (89). This intracellular crawling appeared to be HSEC-specific as it did not occur in more conventional vascular endothelial cells (HUVEC; human umbilical vein endothelial cells). It was subsequently speculated that this process could represent a liver-specific method of immune surveillance (89); however, studies of this phenomenon were all undertaken *in vitro* and it is yet to be confirmed *in vivo*. Interestingly, once leukocytes have traversed the endothelial barrier, they have also been shown to undergo sub-endothelial crawling (90–92) prior to their migration into the tissue proper.

#### Transendothelial Migration

The final step in the leukocyte adhesion cascade is the crossing of the endothelial barrier, which is known as transendothelial migration (93). Transendothelial migration of leukocytes is a highly regulated process as maintenance of barrier integrity is paramount and endothelial cells undergo significant cytoskeletal remodeling to facilitate the passage of leukocytes, whilst also preventing vascular leakage (94). There are two possible routes for leukocytes to transmigrate the endothelial barrier, the paracellular pathway, or the transcellular pathway [reviewed extensively by Ley et al. (35) and more recently by Vestweber (36)]. The paracellular route describes the passage of leukocytes between the cell-cell junctions of the endothelial layer and has inevitably been shown to be mediated via a number of key junctional proteins, such as platelet endothelial cell adhesion molecule (PECAM)-1 (also known as CD31) (95), CD99 (95, 96), and junctional adhesion molecules (JAMs) (97, 98). Also, vascular endothelial (VE)-cadherin has been shown to play an instrumental role in the inhibition of leukocyte extravasation and must be actively moved away from the site of leukocyte transmigration to allow the process to occur (99, 100). The vast majority (~80–95 %) of cells undergo transendothelial migration via the paracellular route; however, the remainder transmigrate through the transcellular pathway which involves leukocytes passing directly through the cell body of endothelial cells This process is highly coordinated and requires extensive remodeling of the endothelial cell's actin cytoskeleton to form an appropriately sized pore to accommodate the passage of the leukocyte, and in particular its nucleus (101). Unsurprisingly, the transcellular migration of leukocytes is stringently regulated by the endothelial cell to minimize vascular leakage (101). The molecules involved in transcellular are less well-studied than those for paracellular migration; nevertheless, to date, ICAM-1 (53, 94, 102, 103) has been identified as the major contributor, but other molecules, such as PECAM-1, JAM-A, and CD99 (104, 105) have also been shown to play a role in this process.

With the technological advancements in microscopy, our knowledge of the processes involved in leukocyte transmigration are ever-increasing (94, 101, 106, 107); nevertheless the molecular mechanisms which determine whether leukocytes transmigrate through the paracellular or transcellular pathways still remain a mystery. The possibility of scavenger receptors playing a role in these processes is a tangible prospect and should be investigated in future studies.

## Scavenger Receptors in Leukocyte Trafficking

Given that a number contain similar structural domains to those found in the selectin family [e.g., C-type lectin domains or epidermal growth factor (EGF)-like domains], it is perhaps unsurprising that several endothelial-expressed scavenger receptors are also able to directly bind to leukocytes. Consequently, several scavenger receptors have been shown to play a role in leukocyte trafficking through lymph nodes and/or in their extravasation through a range of endothelia. Discussed below are the scavenger receptors identified to date which play a role in these processes.

### SR-AI

Scavenger receptor (SR)-AI, also known as macrophage scavenger receptor (MSR)-1 or CD204, was the first scavenger receptor to be cloned (108), and hence is the first member of the Class A family and arguably the most studied scavenger receptor (109). SR-AI is a Type II membrane protein, with its structure consisting of a short N-terminal cytoplasmic tail, a transmembrane domain, a spacer region, an α-helical coiled-coil domain, a collagen-like domain, and a C-terminal scavenger receptor cysteine-rich (SRCR) domain (110). As is characteristic of most scavenger receptors, SR-AI has been shown to bind a highly diverse range of endogenous products including: an array of modified LDLs (111, 112); apoptotic cells (113); heat shock proteins (Hsp) (114); collagen (115); β-amyloid (116); apolipoproteins (117), and advanced glycation end products (AGE) (118). Additionally, SR-AI can also bind a range of exogenous ligands, such as bacterial lipopolysaccharide (LPS) (119), and lipoteichoic acid (LTA) (120), fungal β-glucan (121), and viral double stranded (ds)RNA (122–124). SR-AI is predominantly expressed in myeloid cells, such as monocytes and tissue-resident macrophages, but was also shown to be expressed in high endothelial cells of postcapillary venules (HEV) in peripheral lymph nodes a number of years ago (125). The adhesive ability of SR-AI has only recently been considered; however, this recent study has focused on lymphocyte binding to lymphatic endothelial cells (LEC) (126). In their investigation of SR-AI in LEC, Iftakhar-E-Khuda et al. utilized binding assays to primary murine lymphatic endothelial cells *in vitro* and antibody blockade on human and murine lymphatic tissue sections *ex vivo* to demonstrate its lymphocyte binding capacity in afferent lymphatics (126). However, they did not observe any differences in lymphocyte populations in the lymph nodes of wild type (WT) and SR-AI^−/−^ mice, possibly suggesting a possible redundancy in SR-AI's lymphocyte binding activity *in vivo*, under homeostatic conditions. This discrepancy between the *in vitro* and *in vivo* data suggests that further investigation of SR-AI's adhesive properties is warranted and future studies could possibly explore lymph node trafficking of leukocytes in mice subjected to injury, such as LPS-induced toxemia. Additionally, given the SR-AI expression in HEVs and that inducible expression of SR-AI has been found in human arterial endothelial cells (127), it is not unreasonable for future investigations to explore SR-AI expression in a range of vascular endothelia from different tissues. If found in these vascular endothelial cells, basic static and flow-based adhesion assays, such as those utilized previously in our lab (128), could be employed to determine which step in the leukocyte adhesion cascade SR-AI potentially acts. Furthermore, a leukocyte-expressed ligand has yet to be explored and so future studies should also aim to identify the molecule(s) involved in SR-AI-mediated leukocyte binding to these endothelia.

### LOX-1

Lectin-like oxidized low-density lipoprotein receptor-1 (LOX-1) is another Type II membrane protein which comprises of a short N-terminal cytoplasmic domain, a single transmembrane region and an extracellular domain containing a coiled-coil “neck” region and a C-type lectin-like domain (129) and was the first member of the Class E family to be described. As its name suggests, LOX-1 was initially identified as a receptor for oxLDLs in endothelial cells (129), but has since been shown to bind a number of other modified LDLs, such as carbamylated LDLs (130) and glycoxidised LDLs (131). Subsequently, LOX-1 has also been found to bind a more diverse range of ligands, including phosphotidylserine on apoptotic cells (132, 133), Gram positive and Gram negative bacteria (134), and C-reactive protein (CRP) (135). Nevertheless, LOX-1 is a “non-essential” protein, as LOX-1^−/−^ mice do not exhibit any phenotypic traits. Also, under physiological conditions, LOX-1 is expressed in relatively low levels in vascular endothelial cells, but is inducible upon endothelial activation by ligand binding (136, 137), inflammatory cytokines (138, 139) or shear stress (140). The leukocyte adhesive ability of LOX-1 was first described in 2002, when Hayashida et al. demonstrated that transfected Chinese hamster ovarian (CHO) cells over-expressing LOX-1 augmented the adhesion of primary peripheral blood mononuclear cells (PBMCs), and monocytic cell line, THP-1, when compared to control transfected cells (141). Interestingly, this effect appeared to be monocytic cell-specific, as they did not observe any effects on the Jurkat leukaemic T cell line (141). Additionally, they demonstrated that the enhanced adhesion of THP-1 cells to the LOX-1-CHO could be reversed by antibody or oxLDL blockade and recapitulated this blockade on bovine aortic endothelial cells (BAEC) *in vitro* (141). Finally, they demonstrated that THP-1 cells flowed over LOX-1-CHO cells at increasing shear stress exhibited increased numbers of cells rolling and at lower rolling velocities than those flowed over WT-CHO cells (141), thus suggesting that LOX-1 acts as an adhesion molecule in the early stages of the leukocyte adhesion cascade.

Following this initial study, Li et al. then demonstrated that antibody blockade of LOX-1 *in vivo*, in a rat myocardial ischaemia-reperfusion model, was able to significantly reduce the number of infiltrating leukocytes to the myocardial tissues, which also resulted in a significant decrease in the myocardial infarct (142). However, their data suggested that the diminished leukocyte infiltration was due to an indirect effect of LOX-1 blockade, as they showed a reduction in the expression of adhesion molecules, such as ICAM-1, VCAM-1, and P-selectin (142). Nevertheless, in a seminal study, Honjo et al. demonstrated in a rat model of endotoxemia and endotoxin-induced uveitis, that antibody blockade of LOX-1 expression induced in retinal endothelial cells significantly reduced the number of rolling infiltrating leukocytes, which predominantly consisted of neutrophils, and also increased the velocity of rolling (143). This data is suggestive of a direct interaction with leukocytes *in vivo* and adds to *in vitro* studies which show that LOX-1 functions as adhesion molecule in the early stages of the leukocyte adhesion cascade. Also, more recently, Ding et al. demonstrated that LOX-1^−/−^ mice fed a high cholesterol diet exhibit a lower level of macrophage accumulation in their aortas compared to WT mice (144); nevertheless, it is unclear whether this was due a lack of LOX-1-mediated recruitment by the aortic endothelial cells or a migratory defect in the LOX-1-deficient macrophages.

From the current data implicating it in the leukocyte adhesion cascade, it is clear that LOX-1 contributes to the rolling stage of the adhesion cascade in the recruitment of myeloid cells to a range of vascular endothelia. Nevertheless, despite a number of studies now demonstrating this both *in vitro* and *in vivo*, the leukocyte-expressed ligand(s) responsible for LOX-1 binding have not yet been identified. Additionally, initial studies have suggested that the adhesive properties of endothelial-expressed LOX-1 do not extend to cells of lymphoid lineage, this has only been tested utilizing a leukaemic T cell line and so further investigation with primary lymphocytes could in fact be warranted.

### Mannose Receptor

The third member of the Class E scavenger receptor family to be described, the mannose receptor (MR) or CD206, is a Type I membrane glycoprotein which consists of a short intracellular domain, a transmembrane domain, and an extracellular region comprising of a eight C-type lectin-like domains, a fibronectin type II domain and an N-terminal cysteine-rich domain (145). As its name suggests, MR was originally discovered to bind mannose and other carbohydrate groups in a range of glycoproteins (146); nevertheless, given that its extracellular region comprises of several functionally distinct domains, MR has since been shown to bind a wide range of other endogenous ligands, including collagen (147, 148), CD45 (149), tumoural mucins (150), and neutrophil-derived myeloperoxidases (151). Additionally, MR can bind a range of bacterial- (152, 153), viral- (154–157), fungal- (158–161), and parasite-derived (21) antigens. The mannose receptor is predominantly expressed by macrophages (162, 163), but has also been described in a range of endothelial cells, such as hepatic sinusoidal endothelial cells (HSEC) (89, 164), dermal endothelial cells (165) and lymphatic endothelial cells (LEC) (166–168). The leukocyte adhesive properties of MR were first described by the Jalkanen group based at University of Turku, Finland in 2001, when they suggested that MR plays a role in lymphocyte exiting from lymph nodes as their data confirmed the MR-mediated adhesion of lymphocytes to LECs (167). These studies also demonstrated that L-selectin, was the lymphocyte-expressed ligand required for MR-mediated static adhesion of lymphocytes to LECs *in vitro*, which the authors believed to most accurately mimic physiological conditions within lymph nodes *in vivo* (167). Further studies by the same group demonstrated the binding of B lymphoblastoid cell lines to LEC and high endothelial venules (HEVs) both on tissues sections *ex vivo* and on isolated cells *in vitro* (166), further strengthening the evidence for the adhesive functionality of MR. Subsequently, these *in vitro* findings were corroborated with *in vivo* experiments by Marttila-Ichihara et al. who demonstrated that the adhesion of both normal lymphocytes and tumor cells to afferent lymphatic vessels was significantly reduced in MR-deficient mice, compared to WT (168). More recently, the Jalkanen group also showed that L-selectin-negative leukocytes trafficking to the lymph nodes utilize CD44 to bind to MR expressed on LECs and subsequently migrate to draining lymph nodes (169). The authors also suggest that therapeutic targeting of MR on LEC could selectively reduce leukocyte migration from the periphery into the draining lymph nodes thus potentially acting to dampen inappropriate inflammatory reactions (169). Expression in vascular endothelial cells, such as HSEC, suggests that MR could also potentially facilitate leukocyte binding in the adhesion cascade and future studies could investigate this.

### SCARF1

Scavenger receptor class F, member 1 (SCARF1 or SR-F1), also known as scavenger receptor expressed by endothelial cells (SREC)-I, was first identified in cDNA libraries from human umbilical vein endothelial cells (HUVEC) (170). SCARF1 is a type I membrane protein which comprises of several extracellular EGF-like domains, a transcellular domain and, unusually for a scavenger receptor, a long serine- and proline-rich cytoplasmic tail (171). SCARF1 has been shown to bind modified low density lipoproteins (LDLs), specifically acLDLs (172), and acts as an endocytic receptor for a wide range of damage-associated products (173), including heat-shock proteins (Hsps) (174–176) and apoptotic host cells via phosphotidylserine-bound C1q protein (7). SCARF1 has been shown to play a key role in the prevention of autoimmunity, as SCARF1-deficient mice spontaneously develop systemic lupus erythematosus (SLE) due to the severely impaired clearance of apoptotic cells in the spleen (7). In addition to binding and internalizing a diverse range of endogenous proteins, SCARF1 also binds a wide array of viral (29, 177, 178), fungal (179), and bacterial (28, 30, 180, 181) antigens and SCARF1 expression in alveolar macrophages has been shown to play an important role in immunological responses to fungal lung infection (179). SCARF1 is also expressed in murine splenic endothelial cells (179) and liver sinusoidal endothelial cells (178) and our lab has corroborated this and recently described for the first time the expression on SCARF1 in primary human hepatic sinusoidal endothelial cells (HSEC) (182). Subsequently, utilizing a combination of flow-based adhesion assays with immobilized recombinant proteins, HSEC, and siRNA silencing in HSEC, we were able to robustly demonstrate that SCARF1 plays a role in the selective recruitment of CD4^+^ T cells to the sinusoidal endothelium under physiological shear stress (182). Additionally, we showed that SCARF1 facilitates this process via the formation of adhesive cups which were also rich in ICAM-1 and F-actin and proposed that SCARF1 acts in the firm adhesion step of the leukocyte adhesion cascade (182). However, we did not explore the possibility SCARF1's involvement in the transendothelial migration step and future investigations from our lab will explore this. SCARF1 is known to form moderate homophilic interactions (183); however, we ruled out the possibility of these interactions in this context, as CD4^+^ T cells do not express SCARF1 (182). Therefore, the lymphocyte-expressed ligand of SCARF1 is yet to be identified and screening experiments could be employed to determine this in future investigations.

### SR-PSOX

Scavenger receptor that binds phosphatidylserine and oxidized lipids (SR-PSOX) is the only member belonging to the class G family of scavenger receptors to date (184) and is structurally unique within the scavenger receptor superfamily. SR-PSOX is a type I transmembrane glycoprotein with its N-terminal extracellular domain, consisting of a CXC chemokine motif and a mucin-like stalk, linked to a transmembrane domain and a short C-terminal intracellular domain (185). SR-PSOX also exists in a soluble form which is shed or enzymatically cleaved from the cell surface via a disintegrin and metalloproteinase (ADAM)-10 and ADAM-17 (186–189). SR-PSOX was first identified in the human monocytic cell line THP-1 and was shown to bind and internalize oxLDL and phosphatidylserine (190). Subsequently, SR-PSOX has also been shown to bind eryptotic erythrocytes (191, 192) and bacterial antigens (193, 194) and has been found to be expressed in a wide range of cell types, including macrophages (195), DCs (196), smooth muscle cells (197), and endothelial cells (189, 198, 199). Early cloning studies on a chemokine known as CXCL16 (200, 201) found it to be structurally identical to SR-PSOX and, as CXCL16 is a highly specific ligand for the chemokine receptor CXCR6, it was soon discovered that SR-PSOX was able to support the adhesion of a range of CXCR6^+^ leukocytes (202–205). Subsequent to these findings, it was suggested that SR-PSOX acts in the “arrest” stage of the adhesion cascade by triggering the conformational activation of β_1_ integrins on leukocytes (206).

Possibly the best studied role for SR-PSOX in the recruitment of leukocytes is in the context of hepatic inflammation (207), with its endothelial-expressed form known to interact with intrahepatic CXCR6^+^ immune cells, such as effector T cells (206, 208), natural killer (NK) cells (209, 210) and NKT cells (199). It has recently been shown that genetic deficiency of SR-PSOX in mice inhibits the extent of inflammation in a model of acetaminophen (APAP)-induced acute liver injury (211). In addition, pharmacological intervention with neutralizing antibodies raised against SR-PSOX has shown inflammation-reducing efficacy in preclinical murine models of sepsis-mediated (212, 213) and carbon tetrachloride (CCl_4_)-mediated (207) acute liver injury. Conversely, an elegant study by Ma et al. has recently shown that HSEC-expressed SR-PSOX plays a key role in the recruitment of anti-tumourigenic NKT cells to the liver in a number of murine models of primary and metastatic hepatic cancers (214). Thus, the therapeutic targeting of SR-PSOX to inhibit hepatic inflammation must be carefully considered with regards to context of the inflammatory injury being treated.

### Stabilin-1

Stabilin-1 is a highly conserved type I transmembrane protein and was the first member of the Class H family of scavenger receptor to be described. It was originally described in 1991 as MS-1 antigen, when it was used as a histological marker for non-continuous splenic sinusoidal endothelial cells (215). Subsequently, three labs independently described the same molecule as FEEL-1, due to its structure containing fasciclin (FAS), epidermal growth factor (EGF)-like, laminin-type EGF-like, and link domains (171), stabilin-1 (216) and common lymphatic endothelial and vascular endothelial receptor (CLEVER)-1 (217); however, due to its official gene nomenclature, *STAB1*, stabilin-1 is increasingly utilized in the literature. An early indicator of stabilin-1's capacity as a scavenger receptor was its constitutive expression in the professional scavenging cells of the non-continuous sinusoidal endothelia in the spleen (215), lymph nodes (218, 219), and liver (220). Interestingly, stabilin-1 expression is also inducible in continuous endothelia, in response to angiogenic and proinflammatory stimuli (221). This inducible expression is thought to originate from the transient non-continuous state that vascular endothelial cells transition through during the rapid growth of blood vessels throughout the wound healing process, tumor vascularization, and chronic inflammatory skin conditions, such as psoriasis. As is a prerequisite of being classified as a “scavenger receptor,” stabilin-1 has been shown to bind a wide variety of ligands, such as: modified LDLs (13, 222); phosphotidylserine expressed on apoptotic cells (223–225); secreted protein acidic and rich in cysteine (SPARC) (226); placental lactogen (227) and microparticles from both Gram positive and Gram negative bacteria (228).

Additionally, a number of early studies showed stabilin-1 to be a multi-functional scavenger receptor with the ability to directly interact with leukocytes and effectively act as a leukocyte adhesion molecule. However, the ability of stabilin-1 to perform this particular function has historically been considered a contentious issue (229, 230), which is possibly confounded by the fact that the leukocyte-expressed ligand(s) for stabilin-1 still remains unidentified. Nevertheless, there is a growing body of evidence for this adhesive function and its first description was by the Jalkanen group (217), when they demonstrated that antibody blockade of stabilin-1 on high endothelial venules (HEVs) and lymphatic vessels, in both *in vitro* static adhesion assays and under flow conditions *in vivo*, significantly diminished the number of adherent lymphocytes, granulocytes, and monocytes (217). Around this time, the same group presented further evidence, showing the stabilin-1-mediated adhesion of B lymphoblastoid cell lines to lymphatic endothelial cells and HEVs *in vitro* (166). Subsequently, this group then demonstrated that stabilin-1 plays a key role in the transmigration of leukocytes through vascular and lymphatic endothelial cells *in vitro* (218) and later confirmed *in vivo* that it mediates the transendothelial migration of T cells and B cells across HEVs to the draining lymph nodes (219). Furthermore, they also showed that antibody blockade of stabilin-1 effectively inhibited peritonitis in mice by decreasing granulocyte recruitment by ~50%, whilst migration of monocytes and lymphocytes into the inflamed peritoneum was almost completely inhibited (219). More recently, the Jalkanen group have also shown that stabilin-1 plays a key role in the recruitment of immunosuppressive macrophages and T regulatory (T_reg_) lymphocytes in *in vivo* models of tumor growth and metastasis, with reduced numbers of both cell types demonstrated in the absence and therapeutic blockade of stabilin-1 (231).

In addition to this, and consistent with the Jalkanen group's data, our lab has implicated stabilin-1 in the transendothelial migration of both T_regs_ and B-cells through hepatic sinusoidal endothelial cells (HSECs) *in vitro*, under conditions which mimic the physiological flow and proinflammatory microenvironment of the hepatic sinusoids during liver injury (89, 220, 232). Interestingly, in the context of hepatic microvasculature, monocyte recruitment does not appear to be supported by stabilin-1, with antibody blockade on HSEC *in vitro* exhibiting no effect on neither monocyte adhesion nor transmigration, under physiological flow (unpublished data). Also, the leukocyte adhesion function of HSEC-expressed stabilin-1 appeared to be redundant *in vivo*, in murine models of liver injury, as no significant differences in T_reg_ or B cell numbers were found between stabilin-1^−/−^ mice and their wild type counterparts, in both carbon tetrachloride (CCl_4_)- and methionine and choline-deficient (MCD) diet-induced liver injury models (13). Nevertheless, given that Karikoski et al. showed significantly decreased numbers of T_regs_ were present in their murine tumor models when stabilin-1^−/−^ mice were compared to WT controls (231), it can be speculated that stabilin-1's role in the recruitment of T_regs_ across HSEC will be potentially important in the context of hepatocellular carcinoma (HCC). Karikoski et al. also showed that stabilin-1^−/−^ mice presented with smaller primary and metastatic tumors than WT mice (231) and these findings were corroborated with preliminary data in human HCC tissues *ex vivo*, which has shown that stabilin-1 expression is highly augmented in peritumoural endothelia and correlated with adverse histological features (233). This suggests that stabilin-1 potentially plays an adverse role in malignancy by potentiating the suppression of the host immune response to a neoplasm; consequently, a Phase I/II trial, TIETALC, (Tumor Immunity Enabling Technology Against Liver Cancer) is currently being designed at the University of Birmingham to test the efficacy of targeting stabilin-1 in HCC (234).

### Stabilin-2

The second member of the Class H scavenger receptor family, stabilin-2, also known as FEEL2 or HARE (hyaluronan receptor for endocytosis), is very similar in structure to stabilin-1 with both exhibiting similar domain organization in their extracellular regions. Stabilin-2 was originally described as a clearance receptor for hyaluronan (216, 235, 236); however, it is now known to bind a wide range of structurally diverse ligands. For example, stabilin-2 has also been shown to bind to acLDLs (228), heparin (237), apoptotic (238, 239), and necrotic (240) cells and microparticles from both Gram positive and Gram negative bacteria (228). Like stabilin-1, stabilin-2 has also been shown to be expressed in HSEC (235, 241, 242) and can also mediate lymphocyte recruitment to the hepatic sinusoidal endothelium (241). Through a number of mutation experiments and antibody blockade studies *in vitro*, Jung et al. found that the fasciclin 1 (FAS1) domains of stabilin-2 were response for lymphocyte binding and identified α_M_β_2_ integrin as the lymphocyte-expressed ligand (241). They also determined that stabilin-2 expression was not regulated in HSEC by proinflammatory stimuli previously shown to activate endothelia and subsequently suggested that stabilin-2 predominantly acts in the firm adhesion step of the leukocyte adhesion cascade as its forced down regulation via shRNA treatment did not affect lymphocyte rolling or transendothelial migration, but was still able to significantly reduce the number of adherent cells (241). Despite their identification of the lymphocyte-expressed ligand for stabilin-2, the study undertaken by Jung et al. remains the only exploration of stabilin-2's lymphocyte binding ability to date. Since monocytes (243) and neutrophils (244) also express α_M_β_2_, it would be interesting to investigate whether or not stabilin-2 is also able to mediate the binding of these myeloid populations. Furthermore, the Jung study was restricted to stabilin-2-mediated lymphocyte binding in the context of HSEC (241); however, splice-variants have also been identified in non-continuous sinusoidal endothelia of other tissues, such as lymph nodes and the spleen (235, 245) and so future studies could also explore the potential role of stabilin-2 in leukocyte recruitment to these alternative tissues.

## Future Work and Therapeutic Potential

Trafficking of leukocytes represents the fundamental basis of any type of immunological response and so targeting this process remains an attractive prospect in the suppression of a wide variety of inflammatory diseases. Whilst many of the key players in this process have been identified, we have summarized the gathering evidence that scavenger receptors can act as atypical adhesion receptors which contribute to leukocyte homing (Figure 1). In summarizing this literature, it is evident that further work is required to understand the exact mechanisms by which scavenger receptors contribute to leukocyte adhesion and migration.

Scavenger receptors can rapidly recycle from the cell membrane (246) and are also known to interact with other pattern recognition receptors (20); this therefore leads to the question of whether or not scavenger receptors contribute to leukocyte adhesion in a direct or indirect manner. In addition, given that scavenger receptors have important homeostatic functions in the remove of endogenous waste products from cell turnover, further experimental work is required to understand how the multifunctional properties of these receptors influence their *in vivo* contributions. It is currently unclear if there is a hierarchy in ligand recognition/affinity and how the leukocyte homing properties of scavenger receptors work alongside their homeostatic functionality. Whilst the experiments described in this review have confirmed a role for scavenger receptors in leukocyte homing, in several cases the identity of the ligand they bind on leukocytes have not been elucidated (Table 1), although imaging has demonstrated that some these receptors, such as stabilin-1 and SCARF1, directly interact with leukocytes on the endothelial surface (182, 220). The development of high resolution imaging will hopefully help answer some of these questions, focusing on the trafficking of scavenger receptors and their membrane dynamics during leukocyte recruitment as well as their interaction with other cell membrane molecules.

**Table 1:** Summary of endothelial-expressed scavenger receptor function, leukocyte/ligand binding, and translational stage of research.

**Scavenger receptor**	**Endothelial cells (EC) studied**	**Role in leukocyte trafficking**	**Leukocyte binding**	**Leukocyte ligand(s)**	**Translational stage**
**LEUKOCYTE ADHESION CASCADE**					
SR-PSOX	Hepatic sinusoidal (HSEC)	Arrest	CD4^+^ and CD8^+^ T cells, NK cells, NKT cells	CXCR6	*In vivo*, murine models of acute liver injury
LOX-1	Bovine aortic endothelial cells (BAEC); rat retinal ECs	Rolling	Neutrophils, monocytes/macrophages	Unknown	*In vivo*, rat model of endotoxemia
SCARF1	Hepatic sinusoidal (HSEC)	Firm adhesion	CD4^+^ T cells	Unknown	*In vitro*, primary human cell models
Stabilin-1	Peritoneal vascular ECs; tumor vascular ECs; hepatic sinusoidal (HSEC)	Transendothelial migration	T_reg_, B cells, granulocytes and monocytes	Unknown	Phase I/II clinical trials in HCC being designed
Stabilin-2	Hepatic sinusoidal (HSEC)	Firm adhesion	PBLs	α_M_β_2_ integrin	*In vitro*, primary human cell models
**LYMPH NODE TRAFFICKING**					
SR-AI	Lymphatic (LEC)		PBLs	Unknown	*In vitro*, primary murine and human cell models *Ex vivo*, static adhesion assays
Stabilin-1	Lymphatic (LEC) and high endothelial venules		T cells, B cells	Unknown	*In vivo*, murine models
Mannose receptor	Lymphatic (LEC) and high endothelial venules		PBLs	L-selectin, CD44	*In vivo*, murine models

Despite the need for further experimental work in this area, the potential of scavenger receptors as therapeutic targets in inflammatory disease should be explored. Due to their enrichment in specialized vascular beds, such as lymphatics and other sinusoidal endothelial vasculature, and the fact that leukocyte recruitment differs here from conventional vascular beds, scavenger receptors may predominantly influence recruitment in an organ-specific manner. They present a promising avenue for the translational development of clinical therapies to target inappropriate inflammatory reactions, such as autoimmunity, as well as hepatic inflammation and recruitment in the bone marrow niche. With regards to the potential targeting of scavenger receptors in the leukocyte adhesion cascade, liver-specific targeting may present more viable therapeutic targets than endothelia of other organs, given the increased expression of scavenger receptors in HSEC (247). Additionally, the low shear stress environment results in a largely selectin-free leukocyte adhesion cascade, thus allowing for a greater contribution by atypical adhesion molecules to leukocyte recruitment.

However, targeting the leukocyte adhesion cascade to treat inflammatory diseases could potentially be associated with significant side effects related to impaired immune surveillance and increased risk of invasion by pathogenic organisms. Nevertheless, detailed analysis of leukocyte recruitment of some scavenger receptors have shown that, rather than a pan-leukocyte effect, some of them influence the trafficking of specific subsets of leukocytes (Table 1). This suggests that these receptors may indeed be therapeutically effective in shaping the immune microenvironment by altering the balance of immune populations at the site of inflammation, whilst also minimizing potential side effects. Stabilin-1, for example, predominantly mediates the recruitment of regulatory T cells across liver endothelium suggesting that blocking its action would be more appropriate in the setting of malignancy to boost tumor-specific immune responses, whilst other scavenger receptors, such as LOX-1, appear to be more pro-inflammatory. Several preclinical experimental approaches have utilized monoclonal antibodies to block the action of this family of receptors in leukocyte recruitment; therefore, the development of humanized therapeutic antibodies appears to be a reasonable approach to target these receptors in the clinic. However, a caveat when using monoclonal antibodies is the probability of off-target effects, considering the differential expression of many scavenger receptors in a range of professional immune cells. Therefore, a more LEC- or HSEC-specific approach, e.g., adenoviral vector (AVV) delivery of siRNA, would perhaps be the most germane approach, as this would feasibly negate any potential off-target effects. Finally, the emerging evidence that scavenger receptors interact with other receptors and their multifunctional properties suggest that, as well as monotherapies, scavenger receptors could also be combined with other therapies, for example TLR-directed treatments, to alter leukocyte trafficking and boost the effectiveness of other therapies which target other arms of the immune response.

## Conclusions

There is an increasing amount of evidence describing the role of endothelial-expressed scavenger receptors in leukocyte trafficking. In this capacity, a number of scavenger receptors are able to directly interact with leukocytes and mediate their passage across a range of endothelia. This secondary function is relatively understudied and further work could lead to novel immunological therapies which could effectively treat inflammatory conditions and contribute to combinatorial approaches to manage these conditions.

## Disclaimer

This paper presents independent research supported by the Birmingham NIHR Liver Biomedical Research Unit based at the University Hospitals Birmingham NHS Foundation Trust and the University of Birmingham. The views expressed are those of the author and not necessarily those of the NHS, the NIHR or the Department of Health.

## Author Contributions

DP conceived the review, wrote, and edited the manuscript. SS wrote the manuscript.

### Conflict of Interest Statement

SS has received a research grant from Faron Pharmaceuticals to design a Phase I/II trial (TIETALC) of the drug “Clevergen” in patients with HCC. SS also reports consulting for Faron Pharmaceuticals. The remaining author declares that the research was conducted in the absence of any commercial or financial relationships that could be construed as a potential conflict of interest.
